# Misperceptions in the Trajectories of Objects undergoing Curvilinear Motion

**DOI:** 10.1371/journal.pone.0036511

**Published:** 2012-05-17

**Authors:** Ozgur Yilmaz, Srimant P. Tripathy, Haluk Ogmen

**Affiliations:** 1 National Research Center for Magnetic Resonance (UMRAM), Bilkent Cyberpark, Ankara, Turkey; 2 School of Optometry and Vision Science, University of Bradford, Richmond Road, Bradford, United Kingdom; 3 Department of Electrical and Computer Engineering, University of Houston, Houston, Texas, United States of America; 4 Center for Neuro-Engineering and Cognitive Science, University of Houston, Houston, Texas, United States of America; Université Paris 5, - CNRS, France

## Abstract

Trajectory perception is crucial in scene understanding and action. A variety of trajectory misperceptions have been reported in the literature. In this study, we quantify earlier observations that reported distortions in the perceived shape of bilinear trajectories and in the perceived positions of their deviation. Our results show that bilinear trajectories with deviation angles smaller than 90 deg are perceived smoothed while those with deviation angles larger than 90 degrees are perceived sharpened. The sharpening effect is weaker in magnitude than the smoothing effect. We also found a correlation between the distortion of perceived trajectories and the perceived shift of their deviation point. Finally, using a dual-task paradigm, we found that reducing attentional resources allocated to the moving target causes an increase in the perceived shift of the deviation point of the trajectory. We interpret these results in the context of interactions between motion and position systems.

## Introduction

Human observers can misperceive the trajectories of moving objects in a variety of situations [1]–[10]. Some of these misperceptions occur even in the absence of eye-movements [8]. Similar misperceptions sometimes occur with static patterns as in MacKay’s “ray” patterns where static straight lines appear distorted [11]. This paper further investigates the distortions in perceived trajectories previously reported [7]–[9]. Our earlier studies of qualitatively described the misperceptions [7]–[8]. The current paper aims to describe the trajectory-misperceptions more quantitatively and to investigate these misperceptions from the viewpoint of distortions of visual space on account of anticipatory responses to motion [12].

The stimulus in [7] consisted of a target dot moving along a bi-linear trajectory (i.e. a dot moving along a straight line for a few hundred milliseconds and then undergoing a change of direction and moving along a straight line for another few hundred milliseconds) in the presence of distractor dots moving along linear trajectories. The distortions reported consisted of a shift in the point of deviation, followed by an initial overestimation of the angle of deviation and a subsequent compensatory underestimation of the angle of deviation, resulting in the perception of an “S” shaped trajectory. These misperceptions are amplified at the blind spot even under conditions which minimize eye-movements, indicating that eye-movements were not primarily responsible for these distortions [8]. Turn-point shifts, typically around 15 arcmin, have been reported for bilinear trajectories undergoing 90° deviations [9]. Here, we systematically investigated the effect that the angle of deviation has on the perceived shift in the point of deviation.

**Figure 1:**
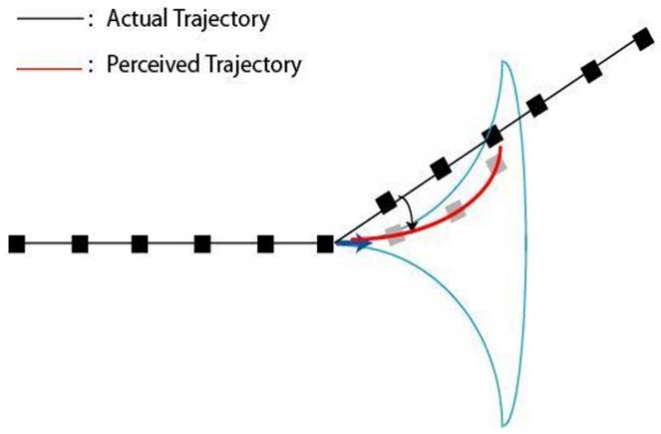
The proposed effect of motion-anticipation on the perceived trajectory. The black squares represent a path of apparent motion and the moving dot deviates from horizontal motion (black line). The motion-anticipation (cone), shifts the perceived positions of the dot resulting in the trajectory shown by the gray dots. The shifts in the perceived positions of the dot causes a curvature in its perceived trajectory (red curve), resulting in smoothing for deviations that are smaller than 90 deg.

Eye-movements are unlikely to explain the trajectory-misperceptions reported in [7]. Questions still remain as to what causes these misperceptions. Tripathy and Barrett proposed an attentional origin of these misperceptions – attention was directed away from the target (on account of the distractors) at the instant when the deviation occurred, causing the detection of the deviation to be delayed; the visual system computed the trajectory without deviation along the straight line until the time that the deviation was perceived. An additional factor that could contribute to the above delay in detecting the deviation is the existence of a perceptual threshold for detecting the deviation – until the normal from the moving dot on to the initial axis of motion exceeds the perceptual spatial-threshold the deviation remains undetected. For example, in Figure 1 the deviation in trajectory remains undetected until the relative vertical height of the dot exceeds threshold (see [13]–[19]). We investigated this shift in perceiving deviations in trajectories and the extent to which attention influences this perceptual shift.

Our previous study used a stimulus consisting of dots that were flashed in the vicinity of moving dots and found that the flashed dots were mis-localized in the direction of the moving dots [12]. These motion-related position illusions were suggested to be caused by anticipatory signals from the motion system that modulate the gain of position-encoding units along the predicted future trajectory of the moving object (see cone in Figure 1). Experimental results indicated that the magnitude of the perceived position shifts was increased if the quality of position cues in the stimulus was reduced. Attention is expected to work counter to the influence of motion and reduce the motion related mis-localizations by enhancing the position encoding signal. Accordingly, in the context of perceived trajectories of moving objects, we propose that anticipation by motion feedback modulates the gain of the position signal and prevents it from exceeding the threshold by attracting it towards the axis of motion before deviation (Figure 1). Hence, an anticipatory influence from motion on the position map is expected to distort the perceived trajectory and shift the point of deviation in the direction of motion (grey dots/line in Figure 1), yielding a perceived trajectory that is curvilinear. Attention is expected to diminish the influence of anticipatory motion signals on the point of deviation by one or more mechanisms. Anticipatory responses to moving stimuli have been reported in retinal ganglion cells of tiger salamanders and rabbits [20], but would presumably be of cortical origin in primates.

In order to investigate the effect of motion anticipation and attention, observers were asked to report the perceived trajectory curvature and perceived deviation point in a bilinear stimulus using a method of adjustment procedure, for trajectories of various shapes. A moving dot deviated in direction in the middle of the screen while observers fixated a fixation cross. After the presentation of the stimulus, observers used a joystick to adjust either the curvature of a line on the screen to report the perceived shape of the trajectory, or a pointer along the horizontal axis to report the perceived deviation point. The fixation cross was replaced by a dual-task stimulus in some sessions to draw away attention from the trajectory of the moving dot.

Experiment 1 investigated the distortions in the perceived trajectories as a function of the angle of deviation of the bilinear trajectories. Experiment 2 investigated the perceived shift in the point of deviation of the dot. Experiment 3 used a dual task to determine the extent to which attention influences the perceived shift in the point of deviation of the dot. These experiments demonstrated that bilinear trajectories are misperceived by the human visual system and that the extents to which the trajectories are misperceived are influenced by the angle of deviation in the trajectory of the moving object and the amount of attentional resources allocated to the object.

Experiments followed a protocol approved by the University of Houston Committee for the Protection of Human Subjects. Each observer gave written consent before the experiments.

## Experiment 1

**Figure 2:**
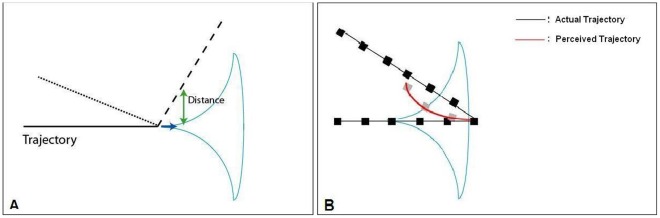
Trajectories and their misperceptions. **A.** Trajectories for two angles of deviation are shown. The solid line is the initial portion of the trajectory, the dashed line represents a 67 deg deviation and the dotted line represents a 157 deg deviation. Motion anticipation is shown as a cone. Its influence is dependent on the distance to the axis of motion after deviation. **B.** The proposed influence of motion-anticipation on the perceived trajectory for angles larger than 90 deg. The black squares represent a path of apparent motion for the moving dot undergoing deviation (black line). The persisting motion-anticipation due to the previous horizontal motion (cone) presumably shifts the perceived positions of the dot closer to the axis of motion resulting in the trajectory shown by the gray dots and red curve. Any shift in the perceived position of the dots would cause the trajectory to appear curved (sharpened).

Anticipation in response to a moving stimulus has been suggested to distort the representation of visual space by modulating the neural activity ahead of the stimulus, leading to misperceptions in the trajectory of the stimulus [12]. We propose that the shift in the perceived point of deviation should be correlated with the distortion in the perceived trajectory. In order to test this hypothesis, we presented trajectories with various angles of deviation and asked observers to report the perceived trajectory. According to the hypothesis, motion anticipation should distort the trajectories for moderate angles of deviation because the influence of anticipation is assumed to be local and dependent on the distance between the axis of motion before deviation and the axis of motion after deviation (Figure 2A). The solid line in the figure represents a trajectory before deviation and the dashed/or dotted lines represent trajectories after the deviation. When the deviation angle is large (dashed line) or the deviation is backwards (dotted line), the influence of motion anticipation on the trajectories, and the resulting distortions, should be small. It should be noted that according to the motion-anticipation hypothesis, for deviation angles smaller than 90 degrees, the post-deviation trajectory will be perceived to be shifted closer to the original direction of motion, resulting in a *smoothing* of the transition (Figure 1), while for deviations larger than 90 degrees, motion-anticipation from the pre-deviation horizontal motion is expected to shift the perceived post-deviation trajectory closer to the original axis of motion, resulting in a *sharpening* of the transition (Figure 2B). Note that smoothing reduces the perceived angle of deviation, whereas sharpening increases the angle of deviation. When the deviation is exactly 90 deg we would expect neither smoothing nor sharpening. A previous study [9] measured the shape of the trajectory with a 90 deg deviation by probing the trajectory at different points; their perceived trajectory (see Figure 5 in [9]) shows distortions from the veridical trajectory, but their plotted trajectory appears bilinear, as would be predicted by the motion-anticipation hypothesis.

### Methods and Stimuli

**Figure 3:**
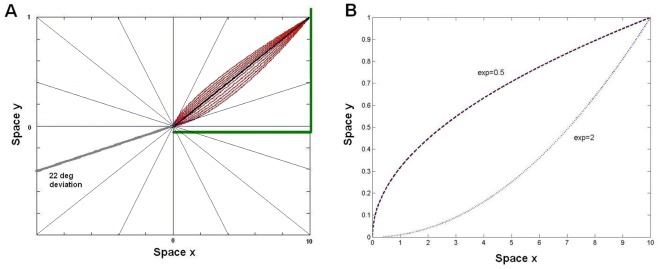
Trajectories used in Experiment 1 and the shapes of different exponents. **A.** A sample trajectory used in the experiment. Space is represented in arbitrary units. The second half of the trajectory was fixed for all deviation angles (45 deg from horizontal, black solid line) but the initial direction of motion was varied according to different deviation angles. The first half of the trajectory for 22 deg deviation is shown by a thick gray line. Observers reported the perceived curvature (curves around the black solid line) in the second portion of the trajectory after each trial. Thick orthogonal lines were placed for reference to facilitate the judgment of the perceived curvature. **B.** The shapes of second halves of trajectories that observers used for reporting their perception, for sample exponent values. The x and y axes represent space, and two different trajectories are shown having exponents of 0.5 and 2. The x axis length is identical for all trajectory shapes and the y axis is computed according to y = y_max_ *(x^/^x_max_)^exponent^, where y_max_ and x_max_ are constants (8.8 arcdeg.). Equivalently we can express the relationship using the differential equation dy/dx = y_max_*exponent*(x_max_)^−exponent^*(x)^exponent−1^.

The stimuli were generated by programming the Cambridge Research Systems (CRS) Visual stimulus Generator (VSG2/5) card and displayed on a 22 inch color CRT monitor. The monitor was set at a resolution of 800×500 with a refresh rate of 160 Hz. The distance between the monitor and the observer was 97 cm at which the screen covered an area of 28 deg×17.5 deg. A head-chin rest was used to stabilize the observers’ head position. Observers fixated a cross (each arm was 21 arcmin) centered 2.1 deg below the middle of the screen and viewed bilinear trajectories that were presented with one of the following angles of deviation: 0 (no deviation), 22, 45, 67, 90, 112, 135, 157 and 180 deg. We anticipated that performance would not be substantially compromised by this eccentric presentation of the trajectories because this eccentricity (2.1 deg) is smaller than the lengths of trajectories used (14.2 deg) in this experiment; since observers fixated a stationary cross, large portions of each trajectory were presented over peripheral retina. In addition, the loss of acuity in amblyopic vision has only a small effect on performance in tasks that used similar stimuli [17]–[18], suggesting that the relatively small loss in acuity at the eccentricity used in the current task is unlikely to have a substantial effect on the pattern of results obtained. Each trajectory consisted of a square dot of side 4 arcmin and luminance 50 cd/m^2^ moving from left to right at a speed of 9.4 deg/sec against a background of 5 cd/m^2^. The direction of motion for the first half of the trajectory varied between trials according to the deviation angle, while that for the second half was always 45 deg from the horizontal (Figure 3A solid black line). The deviation in the trajectory occurred at the center of the screen, after the dot had moved 7.1 deg. Fixing the direction of the second portion of the trajectory for all deviation angles normalized the distortions with respect to a common reference trajectory. Two bars of size 5.7 arcdeg ×4 arcmin (Figure 3A thick orthogonal lines) were placed 21 arcmin below the mid-screen and 5.7 arcdeg to the right of the mid-screen to facilitate judgments of perceived curvature.

After the presentation of the moving dot, a probe line of 6 deg length was presented at the left bottom corner of the screen that represented the second half of the trajectory, i.e. 45 deg from the horizontal. The observers adjusted a parameter called “exponent” with a joystick to report the perceived curvature in the trajectory, ignoring other characteristics of the trajectory. The shapes of the second portion of the trajectories for two different exponents are shown in Figure 3B (mathematical equation provided in the figure caption). An exponent of 1.0 yields a linear trajectory, and misperceived trajectories will be reflected as deviations of exponent values away from 1.0. Phenomenological descriptions obtained from many observers indicated that the exponent is an appropriate parameter for quantifying the perceived trajectories. As a control, trials were interleaved in each session that had actual curvatures in the second half of the trajectory in order to test the observers’ ability to detect and report curvatures. These trials had 0 deg deviations but the exponent value was 1.3 for the second half of the trajectory. Twenty trials were run for each deviation angle and for the control condition, yielding 180 trials for each observer. Three naive observers participated in the experiment.

## Results

On the control trials, the observers were accurate in detecting and reporting the curvature that was physically present in the trajectories. The average reported exponent of 1.33 (with a maximum error of 7%) corresponded closely to the physical curvature with an exponent of 1.3. These results show that our methods for measuring perceived curvature are reasonably reliable, at least for larger exponents. The exponent on the control trials was fixed at the start of the experiment and exceeds unity by approximately two times the largest change in exponent observed in the experiment. In hindsight, a control stimulus with an exponent of around 1.15 would have been closer to the curvatures reported and hence more informative of the observers’ reliability for matching “true” curvatures of the magnitude seen in the current task. However, observers did not report the curvature in the control condition to be an outlier in the current task.

**Figure 4:**
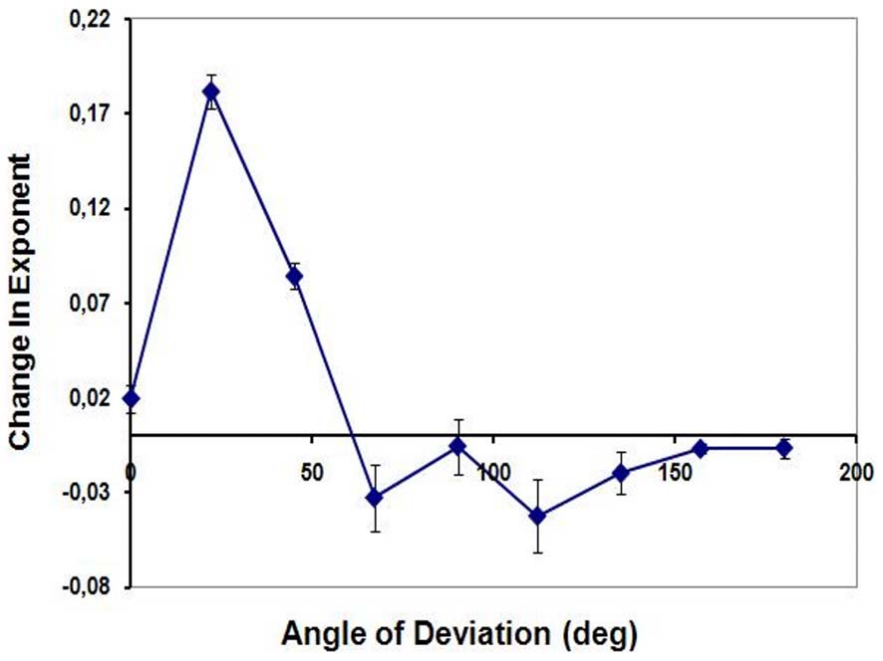
The results of Experiment 1. The average of the three observers for the change in perceived exponents (from actual value 1) of the second half of the trajectory are shown for the 9 deviation angles. Positive exponents indicate smoothing whereas negative exponents indicate sharpening. Error bars correspond to ±1 SEM.

The perceived change in exponent values (from the actual value of 1), averaged across the three observers for each of the 9 deviation angles is shown in Figure 4.

A one-factor repeated measures ANOVA with deviation angle as the independent variable and perceived exponent as the dependent variable was carried out. The results showed a main effect of deviation angle (F[8], [16] = 40.3 p = 0.005, with Greenhouse-Geisser correction for non-sphericity with ε = 0.211). Unplanned pair-wise comparisons with Bonferroni adjustment for multiple comparisons indicated significant differences in exponents for 0 and 22 deg (p = 0.041) and for 22 and 90 deg (p = 0.041); all other unplanned comparisons were not significant.

Visual inspection of the data indicated that, in line with the predictions of the anticipation hypothesis, the measured changes in the exponent for deviations of 0, 90 and 180 deg were close to zero, for deviations between 90 and 180 deg were small and consistent with sharpening and for deviations of 22 and 45 deg were larger and consistent with smoothing. The sharpening seen for deviation of 67 deg was not consistent with the predictions. In order to test the predictions further, each observer’s data for deviations of 0, 90 and 180 were averaged as were his/her data for 22, 45 and 67 deg and for 112, 135 and 157 deg. A one-factor repeated measures ANOVA with the three categories of deviation angles as the independent variable and the averaged exponents as the dependent variable was performed. The results showed a main effect of deviation category (F[2], [4] = 122.07; p<0.001). Pair-wise comparisons with Bonferroni adjustment indicated that exponents for condition [0 deg < deviation <90 deg] were significantly different from those for the condition [90 deg < deviation <180 deg] (p = 0.007) and from those for the condition [deviation  = 0, 90 or 180 deg] (p<0.001). Exponents for the condition [90 deg < deviation <180 deg] were not significantly different from those for the condition [deviation  = 0, 90 or 180 deg] (p = 0.08). The mean changes in exponents were 0.078, −0.023 and 0.003 for the [0 deg < deviation <90 deg], [90 deg < deviation <180 deg], and [deviation  = 0, 90 or 180 deg] conditions respectively.

## Experiment 2

Experiment 1 indicates that trajectories are misperceived when they involve deviations in the direction of motion, and the nature of these misperceptions supports the hypothesis that they might be caused by predictive motion influences. Another aspect of the illusion reported by [7] is a shift in the perceived location of the deviation along the direction of motion (also reported in [9] for a 90 deg deviation). The deviation is expected to be detected by the observers when the moving dot exceeds a spatial threshold in the new direction of motion. Because the proposed motion influence is hypothesized to attract the new axis of motion towards the previous direction of motion (Figure 1), the distortion in the trajectory should be correlated with the shift in the perceived point of deviation. This experiment aims to investigate this correlation and whether a common explanation might link the two distortions.

### Methods and Stimuli

The observers fixated a cross (each arm was 43 arcmin) centered 3.9 deg above the center of the screen and viewed bilinear trajectories that were presented with one of the following angles of deviation: 22, 45, 67, 112, 135, and 157 deg. Each trajectory consisted of a square dot of side 6 arcmin and luminance 50 cd/m^2^ moving from left to right at a speed of 32 deg/sec on a background of luminance 5 cd/m^2^. The dot moved horizontally for a distance of 9 deg, then deviated at the center of the screen. The deviation angle was varied in different sessions.

After the presentation of the moving dot, a probe line 6 deg long was presented at the left-bottom corner of the screen. This line had the same orientation as the second half of the motion trajectory. As in Experiment 1, observers changed the exponent parameter of the probe line to report the perceived shape of the trajectory. Forty trials were run for each deviation angle in different sessions and four observers (two naive) participated in the experiment.

In a separate experiment a probe dot was presented on the horizontal axis after the moving dot disappeared, and the observers adjusted the horizontal position of the dot to report the perceived point of deviation. Again, forty trials were run for each deviation angle and the same observers participated in the experiment.

## Results

**Figure 5:**
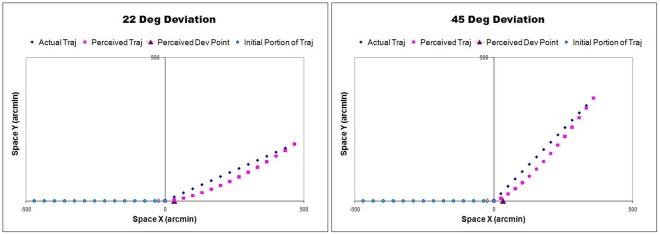
The actual trajectories, perceived trajectories and the perceived points of deviation obtained for observer MA for two of the deviation angles. The axes are in arcmin and the actual trajectories shown are scaled representations of the real stimulus.

The actual trajectories, perceived shape of the trajectories and the perceived point of deviations obtained for observer MA, for the two deviation angles (22 and 45 deg) that yielded the largest misperceptions, are shown in Figure 5; misperceptions for the other deviation angles were smaller. The misperceptions were very similar for the other observers.

**Figure 6:**
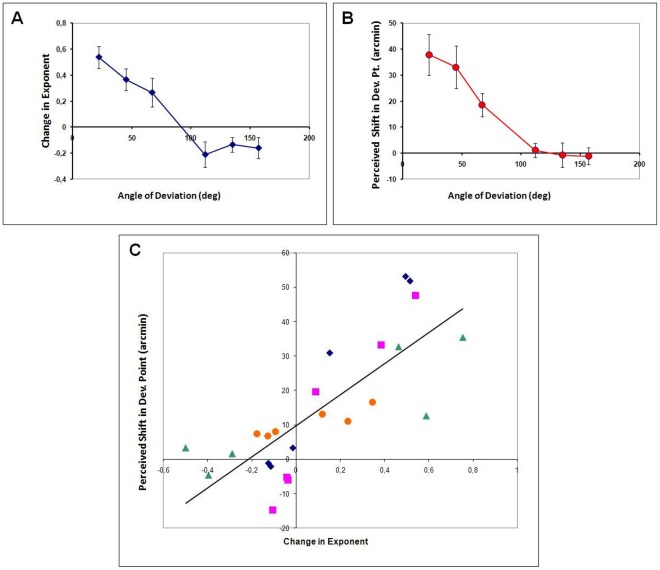
The results of Experiment 2. **A.** The change in the exponent parameter, averaged over the four observers, for the six deviation angles tested. Error bars represent ±1 SEM. Positive exponents for the deviations of 22, 45 and 67 deg indicate smoothing of trajectories whereas the negative exponents for deviations of 112, 135 and 157 deg indicate sharpening. **B.** The shift in the perceived point of deviation (in arcmin), averaged over the four observers, for the six deviation angles tested. Positive values for deviations of 22, 45 and 67 deg indicate a shift of the deviation point in the direction of initial horizontal motion. **C.** The shift in deviation point (in arcmin) is plotted against the change in exponent value for the four observers (indicated by symbols with different shapes). The straight-line fit to the data is also shown (thick diagonal line).

The average change in exponent (difference of the measured exponent from its veridical value of 1) is plotted against the deviation angle in Figure 6A. The results suggest a trend of smoothing (see Figure 1) for deviations smaller than 90 deg and sharpening (see Figure 2) for deviations larger than 90 deg, which is consistent with the previous experiment. A one-factor repeated measures ANOVA with exponent as the dependent variable showed a main effect of deviation angle (F[5], [15] = 11.7 p = 0.022, with Greenhouse-Geisser correction for non-sphericity with ε  = 0.279). Each observer’s three exponent-changes for deviations less than 90 deg were averaged as were his/her three exponent-changes for deviations larger than 90 deg, since these were considered “pseudo-replicates”, as they were not necessarily independent. Two-tailed t-tests were conducted to determine if the means of the averaged change in exponent were significantly different from zero for deviations less than 90 deg and for deviations larger than 90 deg. Smoothing was significant for deviations that were smaller than 90 deg (t(3) = 5.03; p = 0.015; for data for 22, 45 and 67 deg combined) but sharpening was not significant for deviations that were larger than 90 deg (t(3) = 2.19; p = 0.12; for data for 112, 135 and 157 deg combined.

The shifts in the perceived point of deviation for the six deviation angles are plotted in Figure 6B. The perceived deviation point is shifted rightwards in the direction of the initial horizontal motion for deviation angles of 22, 45 and 67. Although some individual observers show perceived leftward shifts for deviations larger than 90 deg, the average values are close to zero. A one-factor repeated measures ANOVA with perceived shift as the dependent variable showed a main effect of deviation angle (F[5], [15] = 10.7, p = 0.032, with Greenhouse-Geisser correction for non-sphericity with ε  = 0.25). Each observer’s three shifts for deviations less than 90 deg were averaged as were his/her three shifts for deviations larger than 90 deg, since these were considered “pseudo-replicates”, as they were not necessarily independent. Two-tailed t-tests were conducted to determine if the means of the averaged shift were significantly different from zero for deviations less than 90 deg and for deviations larger than 90 deg. Positive deviation point shifts were significant for deviations that were smaller than 90 deg (t(3) = 4.368; p = 0.022; for data for 22, 45 and 67 deg combined). However, negative deviation point shifts were not significant for deviations larger than 90 deg (t(3) = –0.386; p = 0.725, for data for 112. 135 and 157 deg combined). When perceived shifts in deviation point are converted into temporal delays by dividing it by the speed of the moving dot, 22 ms of delay is observed for 22 deg deviation and 26 ms delay for 67 deg deviation.

The shifts in deviation points are plotted against the changes in exponent value for the four observers in Figure 6C. The data show a significant correlation between the two variables for the three observers, individually (observer 1 slope = 103.3, p = 0.0003, adj. R^2^ = 0.96; observer 2 slope = 91.5, p = 0.0012, adj. R^2^ = 0.93; observer 3 slope = 30.3, p = 0.027, adj. R^2^ = 0.68; observer 4 slope = 28.6, p = 0.0084, adj. R^2^ = 0.82) or grouped (regression slope = 45.1, p = 0.00001, adj. R^2^ = 0.6). This correlation is suggestive of a common explanation underlying the two misperceptions.

## Experiment 3

**Figure 7:**
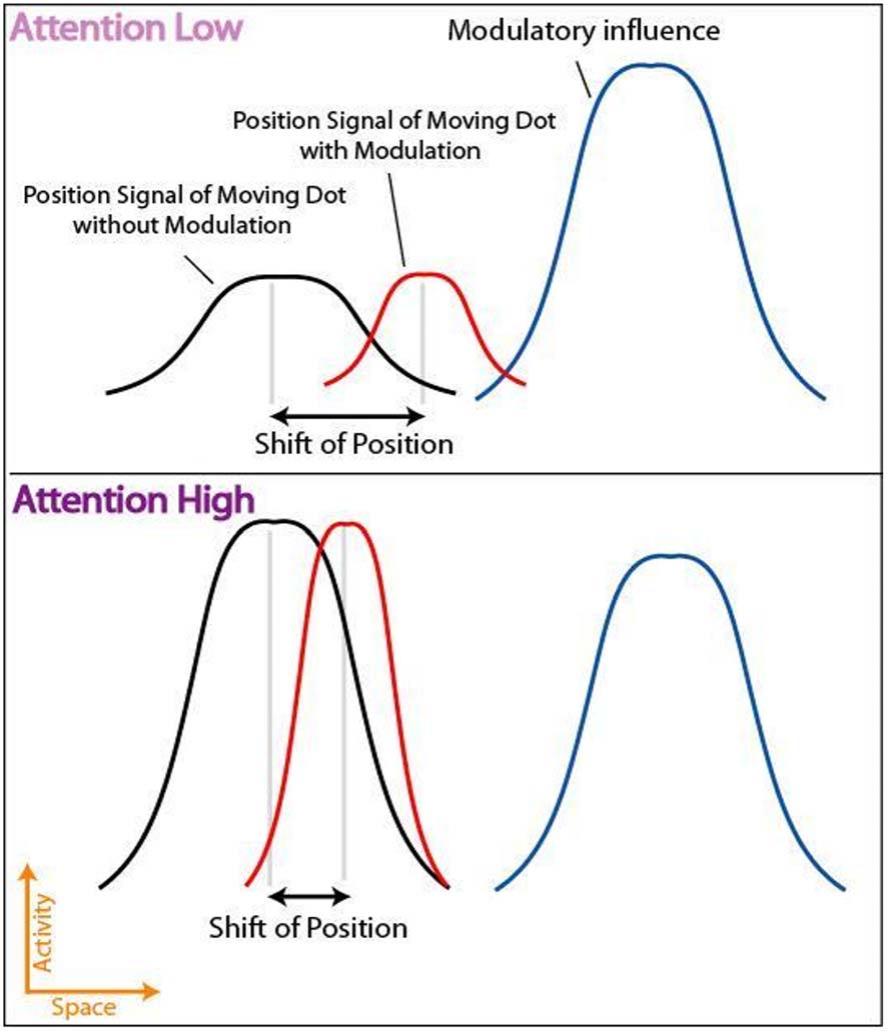
The un-modulated position signal, modulatory influence and position signal after modulation. Two attentional conditions are illustrated: low attention and high attention. When the level of attention directed towards a moving dot is low (upper plot) the dot’s position signal will be weak and spread out, and it will be more prone to perceptual shifts in position due to modulation. In the case where the level of attention directed at a moving dot is high (lower plot), the position signal is stronger, with less spread, which will make it more immune to perceived position-shifts.

As discussed in the Introduction section, attention might be involved in these misperceptions [7]. We propose that attention might increase the neural activity of the neurons in the population, and sharpen the distribution of activities representing attended items in the position map. When attention is focused on the target item, the influence of modulatory signals from motion-sensitive neurons will not shift the position-related neural signals as much as when attentional resources are directed away from the target item and the distribution of the position-map population response is more spread out. The proposition is illustrated in Figure 7. Attention enhances the positional signals of attended items and hence reduces the positional shifts that result from nearby motion. Overall, attention might reduce the motion-induced gain modulation of neurons in the position-map population. The simulations of the model in [12] supported this claim: illusory position shifts of target items increased with the standard deviation of the population activity representing the target items in the position-map.

We used a dual task to reduce the attentional resources available for processing the motion trajectory and measured the resulting misperceptions in the trajectory. We anticipated that reducing the attentional resources would increase the perceived distortions in the trajectories. We asked observers to report the perceived point of deviation of the trajectories from horizontal, which was found to be correlated to the distortion in the shape of the trajectories in Experiment 2. We used trajectories of various shapes to further investigate the spatial characteristics of the motion influence on the perceived shape of the trajectories.

### Methods and Stimuli

The shape of the trajectories, as defined by the exponent parameter (Figure 3B), was varied between sessions. Seven exponents from 0.5 to 2 in steps of 0.25 were used in the experiment. All trajectories in this experiment had a fixed deviation angle of 35 deg. This deviation angle was chosen based on the previous data, which showed substantial misperceptions of the trajectories for deviation angles around 45 deg. Observers reported the perceived point of deviation of the trajectory from horizontal by moving a probe dot using a joystick. In other respects, the methods and stimuli were the same as Experiment 2 except that, in some of the sessions the fixation-cross was replaced with a square of side 43 arcmin and luminance 33 cd/m^2^. In these sessions (called “dual task”), on each trial the luminance of the square either increased or decreased by 2.8 cd/m^2^ for 19 ms, in synchrony with the deviation of the moving dot (which occurred halfway through the trajectory time-wise, with a time-jitter of 40 ms), and observers first reported whether the luminance decreased or increased and then reported the perceived point of deviation. The dual task was expected to reduce the attentional resources allocated to the motion trajectory. The trials with incorrect responses to the luminance task were discarded, and among the trials that remained there were at least 20 in each exponent condition. Three observers (2 naive) participated in the experiment.

**Figure 8:**
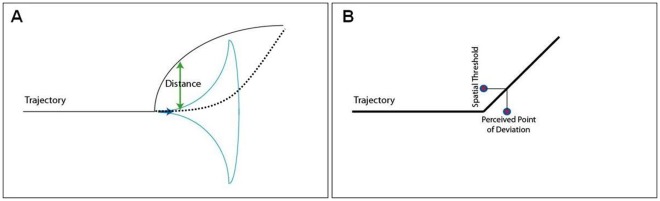
Trajectories with small and large exponents, and spatial threshold calculation from perceived point of deviation. **A.** Trajectory for a small exponent is shown by the solid curve and for a large exponent by the dashed curve. The motion-anticipation is shown as a cone. The cone will not effectively shift the trajectory with the small exponent because of the large distance between them and large exponent trajectory because of the smooth transition in direction. **B.** Estimating the spatial deviation threshold. The ordinate of the trajectory at the perceived point of deviation represents the inferred spatial threshold for detecting the deviation. This is illustrated for a bilinear trajectory.

We anticipated the following effects with regard to the misperception of the trajectories as measured by the perceived point of deviation of the trajectory from horizontal:

- The misperceptions would be smaller when the exponent representing the curvature is small. This is because smaller exponents would take the trajectory further from the cone of influence of motion as illustrated in Figure 8A (solid curve). Also, the influence of motion in inducing misperceptions would be small for very large exponents because in this case the motion is smooth and the cone of anticipation is following the motion as it rotates (Figure 8A dashed curve). The modulation field of the cone is aligned with the trajectory hence the misperceptions caused by the cone are expected to be small.
- The misperceptions would be larger if attentional resources were directed away from the motion-trajectory on account of the dual task.
- The dual task would be most effective in inducing misperceptions when the exponents representing the actual curvature of the trajectories are in between the low and high values of the exponent. This is because the misperception due to motion is maximized for intermediate exponents (as proposed in i).

## Results

**Figure 9:**
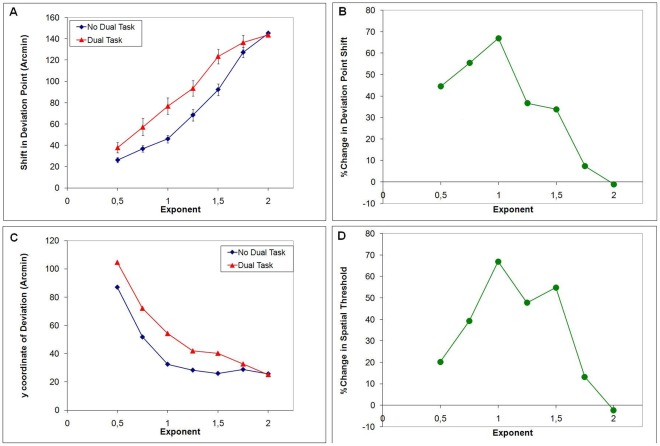
Results of Experiment 3. **A**. Shift in deviation point vs. exponent of the trajectory for two attention conditions. The perceived shift in deviation point monotonically increases with the exponent. Dual task condition has larger shifts in general. Error bars represent ±1 SEM. **B**. The percent change in deviation point shift (%change due to dual task) is plotted against the exponent. The percent increase in the illusory position shifts due to dual task is more pronounced for intermediate exponents. **C.** The spatial thresholds computed from Figure 9A via the formula suggested in Figure 8B. **D.** The percent change in y axis spatial threshold due to dual task. Attention improves the spatial threshold mainly for intermediate deviation angles.

The shifts in the deviation points for the two attention conditions, averaged over the three observers, are shown in Figure 9A as a function of the exponent representing the actual curvature. The misperception of the point of deviation increases systematically with exponent, with or without the dual task (two-way repeated measures ANOVA (7 exponents ×2 task conditions), Effect of exponent: F[6], [12] = 52.9, p = 0.002, with Greenhouse-Geisser correction for non-sphericity). Regression analysis: No Dual task slope = 83.4, p = 0.00001, fixed effect = −12.6, adj. R^2^ = 0.77; Dual Task slope = 74.6, p = 0.00001, fixed effect = 1.03, adj. R^2^ = 0.72). The largest misperceptions in the point of deviation were as large as 140 arcmin of visual angle. The effect of the dual-task was to systematically increase the misperception of the point of deviation as predicted, though this trend was only marginally significant (repeated measures ANOVA, Effect of dual task: F[1], [2] = 16.9, p = 0.054). As a relative measure, the percent change in the deviation point shift in the dual-task condition is plotted in Figure 9B, which shows that reducing the attentional resources available for the processing of the motion trajectory results in the largest relative increase in the misperceptions when exponents are close to linear. The dual task had a maximum effect on the misperceptions when the exponent was 1.0. For this exponent the misperception in the dual-task condition (77 arcmin) exceeded that in the non-dual-task condition (46 arcmin) by 67%. The exponent*task interaction was not significant (F[6], [12] = 1.649; p = 0.317).

The perceived points of deviation in Figure 9A can be converted into vertical offsets of trajectories at the perceived deviation points and these would represent spatial-thresholds (Figure 8B) for detecting the deviation. Spatial thresholds for detecting the deviations are plotted in Figure 9C for the two attentional conditions and percent change are shown in Figure 9D. The largest spatial thresholds were larger than 80 arcmin and under the best of conditions offsets had to exceed 25 arcmin before the deviations could be detected. For an exponent of 1.0, the spatial offset in the dual task condition (54 arcmin) was larger than that in the non-dual-task (32 arcmin) condition by 68%.

## Discussion

How the visual system computes the position of moving objects is largely unknown. The perceived positions, or trajectories, of moving objects are not always veridical (e.g. [7] [12] [21]–[25]) and these misperceptions can be used to probe the underlying computational mechanisms. A commonly used method to measure perceived position of moving objects is to ask observers to compare a target stimulus with a simultaneously presented reference stimulus. The reference stimulus can be itself either a moving stimulus [26] or a flashed stimulus [11] [27]–[29]. Since timing is critical in position judgments for moving stimuli, this method of cross-stimulus comparison may not be ideal since it has been suggested that differential latencies between the target and reference stimuli can affect significantly the magnitude of the illusion [30]–[35]. For an extensive review of models proposed for position computation based on flash-lag data the reader is referred to [35]. Here we used an approach whereby observers report from memory the perceived shape and the point of deviation by a method of adjustment. Thus, our approach provides estimates of how trajectories of moving targets are processed and stored in memory by avoiding the effect of the unknown spatiotemporal dynamics of the reference stimulus.

Our results show that bilinear trajectories with deviation angles smaller than 90 deg are perceived smoothed while those with deviation angles larger than 90 degrees are perceived sharpened. The sharpening effect is weaker in magnitude than the smoothing effect. We also found a correlation between the distortion of perceived trajectories and the perceived shift of their deviation point. Finally, using a dual-task paradigm, we found that reducing attentional resources allocated to the moving target causes a marginal increase in the perceived shift of the deviation point of the trajectory.

The misperceptions reported here are of much smaller magnitude than those reported in [7] and [8]. In these earlier studies the stimuli had been selected so as to enhance these misperceptions. For example, in [7] additional trajectories were added to the stimulus; it is now understood that introducing additional trajectories to the stimulus results in the loss of precision in the internal representation of each trajectory [15]–[16] [19] [36]–[39]. In [8] having the deviations occur within the observers’ blind spots delayed their detection. The current study used single-trajectory, bilinear stimuli without any occlusion of the deviation, but even for these simple trajectories misperceptions were observed. It remains to be seen if the stimulus manipulations used in these earlier studies could enhance the marginal misperceptions of the current study into more robust misperceptions.

When an object appears with a sudden onset, moves for a while, and disappears with a sudden offset, its initial and final positions are typically mislocalised in the direction of motion. These illusions are referred to as the *Fröhlich effect* and *representational momentum* (RM) respectively [40]–[46]. The misperceptions in trajectories reported here are not easily explained by standard versions of any of these illusions for the following reasons:

- In each of the aforementioned illusions, the misperceived locations fall on the actual trajectories, or their extensions. For example, the perceived starting point of the trajectory in the Fröhlich effect is a point on the actual trajectory. The trajectory-curvature can be misperceived only if individual points along the trajectory are perceived to lie away from the actual trajectory.
- Proposed explanations for the Fröhlich effect involve the reduced visibility of the stimulus due to the delay in engaging visuo-spatial attention at stimulus onset and due to metacontrast suppression at earlier locations of the stimulus (e.g. [42]). The current experiments involved reporting the curvature of the second half of each trajectory, with the deviation occurring approximately 750 ms after the onset of motion. Therefore, attention should already be focused on the moving object at the moment of deviation. In addition, there was no obvious reduction in the visibility of the stimulus around the point of deviation.
- Comparison of the current study with the RM studies is complicated by the fact that many of the RM studies (e.g. [43]–[46]) used “implicit motion”, which according to [45] is “a succession of static displays depicting the changing positions of a pattern or its elements that would occur at regular intervals during continuous movement, but without presenting the actual movement”. In contrast to the RM studies the current study used apparent motion stimuli that closely mimicked continuous movement. However, some similarities exist between some of our current findings and earlier RM studies. For instance, the use of a distracting objects or the use of a competing dual-task in RM studies is seen to increase the mislocalisation of the target object in the direction of continued motion [46].

We interpret these trajectory distortions in the context of interactions between motion and position systems. Specifically, we suggest that anticipatory signals, estimated from the current direction of motion, are used to modulate the gain of neurons in the position map in order to prime responses in the likely future direction of the target. While such priming can be useful in enhancing the responses to a moving target (i.e. shorter latency), it may also cause distortions when changes in the trajectories occur. Due to the inertia of priming, when the target changes direction, the trajectory will be distorted towards the predicted trajectory yielding smoothing and sharpening for deviations less than and larger than 90 degrees. These distortions will be prominent especially when trajectory deviation goes undetected. In agreement with this hypothesis, we found a correlation between perceived shifts of deviation points and the magnitude of distortions. A natural extension of these findings is that attention should be able to modulate these distortions by improving the signal encoding the trajectory of the moving target and by facilitating the detection of the point of deviation. We have also shown that directing attention to trajectories can reduce their misperceptions, which we interpret as consequences of an improvement of the trajectory signal and an earlier detection of the deviation point.
